# Neurogenic Bladder-Induced Stone in a Pelvic Kidney of a Caudal Regression Syndrome Patient: Management of a Complex Case

**DOI:** 10.7759/cureus.25479

**Published:** 2022-05-30

**Authors:** Mohammad A Alomar, Mohammad A Alghafees, Abdulaziz Aljurayyad, Hamad S Alsuhaibani, Sultan S Almaiman, Tariq S Alotaibi

**Affiliations:** 1 Urology, King Faisal Specialist Hospital and Research Centre, Riyadh, SAU; 2 College of Medicine, King Saud Bin Abdulaziz University for Health Sciences, Riyadh, SAU; 3 Urology, King Saud University Medical City, Riyadh, SAU; 4 Interventional Radiology, King Faisal Specialist Hospital and Research Centre, Riyadh, SAU

**Keywords:** pelvic ectopic kidney, caudal regression, endo urology, skeletal deformities, kidney stones, percutaneous nephrolithotomy (pcnl)

## Abstract

Percutaneous nephrolithotomy (PCNL) is a difficult treatment for treating kidney stones, especially when there are orthopedic or skeletal abnormalities. Here, in a 19-year-old male, we describe a two-step PCNL with a case of caudal regression syndrome (CRS) and a pelvic kidney, with an extremely deformed neurogenic bladder on intermittent catheterization. Our conclusion is that PCNL may be done safely with minimum morbidity in patients with caudal regression syndrome by utilizing adult equipment for heavy stone burdens, allowing full and rapid stone removal.

## Introduction

Sacral agenesis syndrome and caudal dysplasia are other names for caudal regression syndrome. It's an uncommon congenital abnormality that causes developmental failure in varying degrees early in pregnancy [1]. One to three newborn children per 100,000 live births are affected by CRS [2]. Diabetic mothers' infants have a prevalence of 1 in 350 live births, which covers all varieties [1]. It's a rare congenital condition marked by varying degrees of caudal vertebral agenesis or dysgenesis, as well as spinal cord malformations. Depending on the anatomical degree of their spinal agenesis, patients with CRS have a wide variety of associated changes. Individuals with CRS have a significantly higher rate of renal and genitourinary problems, such as renal agenesis, horseshoe kidney, ureteral duplication, and pelvic kidneys, than patients with other neural tube defects, and they are more likely to have renal function impairment. The severity of the sickness has been found to be inversely associated with the degree of surviving spinal cord function.

Pelvic kidneys are usually found by chance since they are asymptomatic. One in every 2,200-3,000 people is thought to be affected [2]. They might be signs of nephrolithiasis, hydronephrosis, uteropelvic junction blockade, or the formation of calices, among other things. Because neurogenic bladder occurs in 60% of CRS patients, it is critical that all CRS patients be properly tested for urinary obstruction at any part of the tract [2]. The use of a PCNL in a patient with CRS is described in this study. During the surgery, the kidney stone was successfully removed by a PCNL and an antegrade ureteroscopy.

## Case presentation

**Figure 1:**
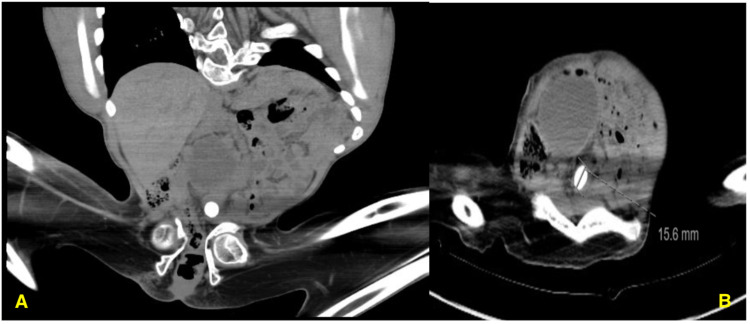
(A) Coronal view of a preoperative CT scan showing a 15.6 mm distal ureteric stone and a pelvic kidney; (B) axial view of a preoperative CT scan showing a 15.6 mm distal ureteric stone and a pelvic kidney with hydronephrosis

In our case, a 19-year-old man was diagnosed with caudal regression syndrome and a neurogenic bladder on intermittent catheterization. He had complaints of on and off flank pain. The Mitrofanoff procedure, a cecostomy surgery, and two unsuccessful ureteroscopic stone removal surgeries are all part of his surgical history. This patient was followed as a case of bilateral hydronephrosis, mainly on the left side, and a kidney stone. His last creatinine level was 49 mg/dl. On computed tomography (CT) scan, there was a left distal ureteric stone of about 1.5cm (Figures 1a-1b). Thus, the patient was scheduled for a percutaneous nephrolithotomy (PCNL) procedure.

**Figure 2:**
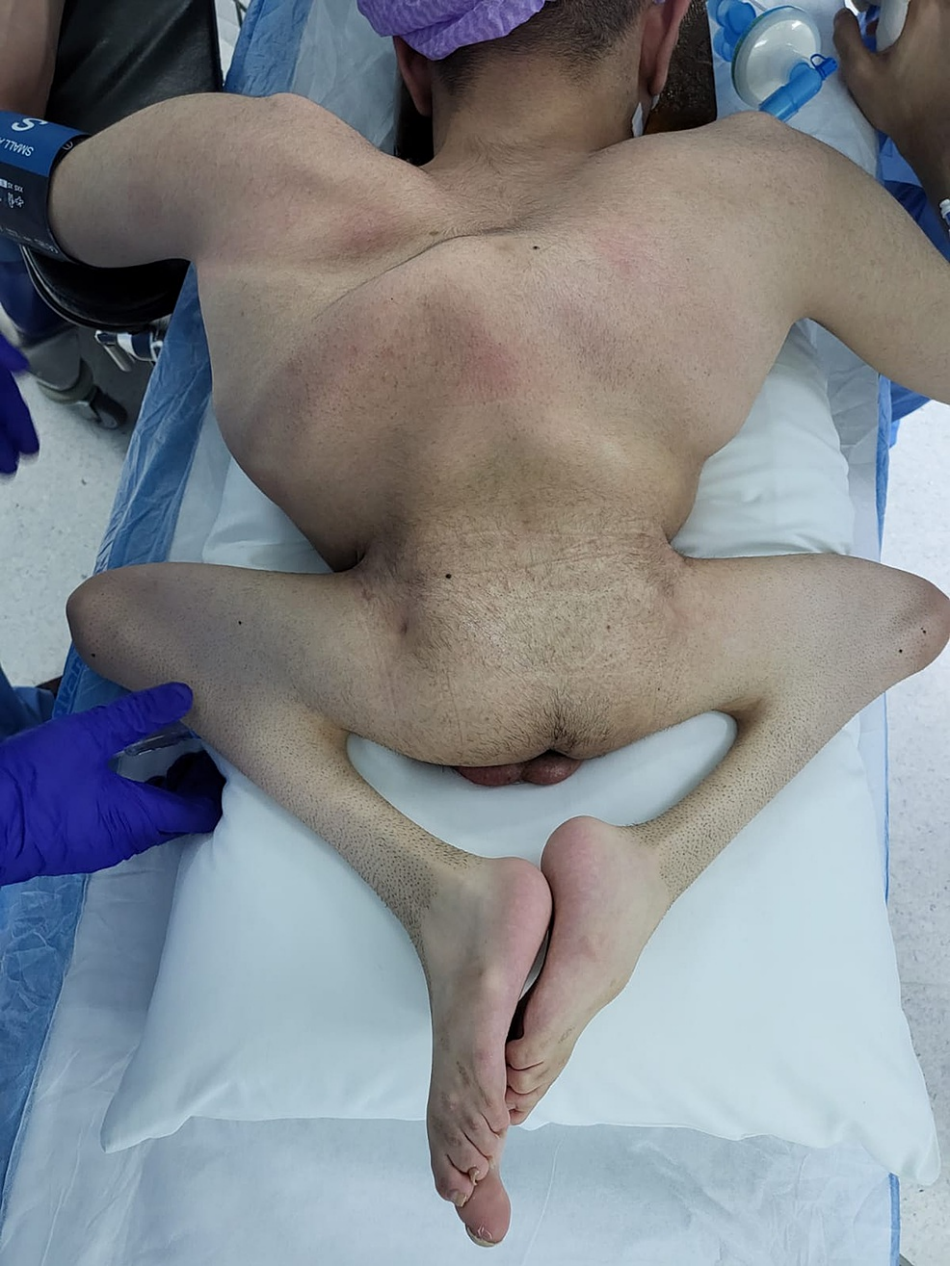
Preoperative image of the patient in a prone position

In the prone position, the patient was intubated, prepped, and draped in a sterile manner (Figure 2).

**Figure 3:**
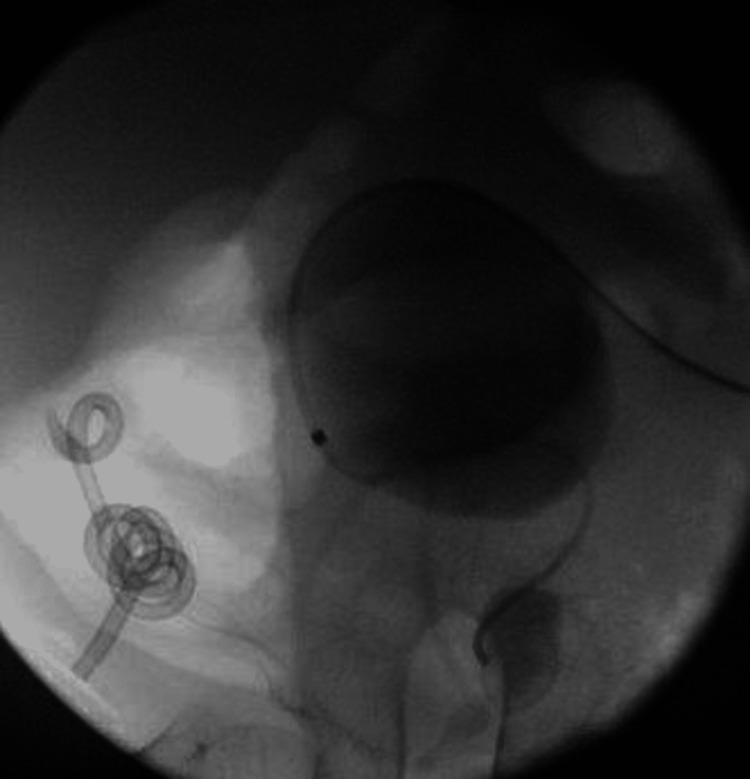
Fluoroscopic image of the hybrid guidewire advanced into the bladder

On the operating table, ultrasound-guided percutaneous access was obtained by the interventional radiologist. Afterward, a hybrid guidewire was advanced into the bladder after manipulation with an angled angiocath (Figure 3). An 8-10 co-axial set was used to advance a second guidewire in the bladder.

**Figure 4:**
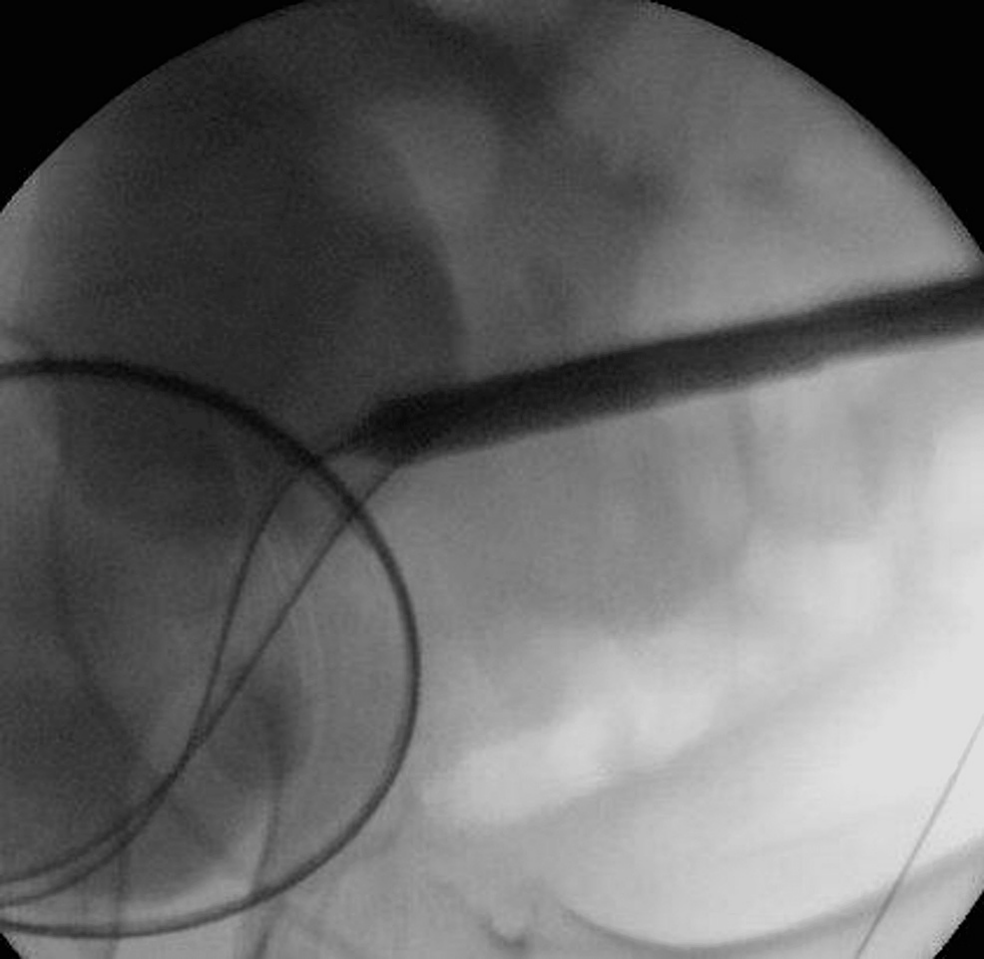
Fluoroscopic image of the nephrostomy balloon dilator

**Figure 5:**
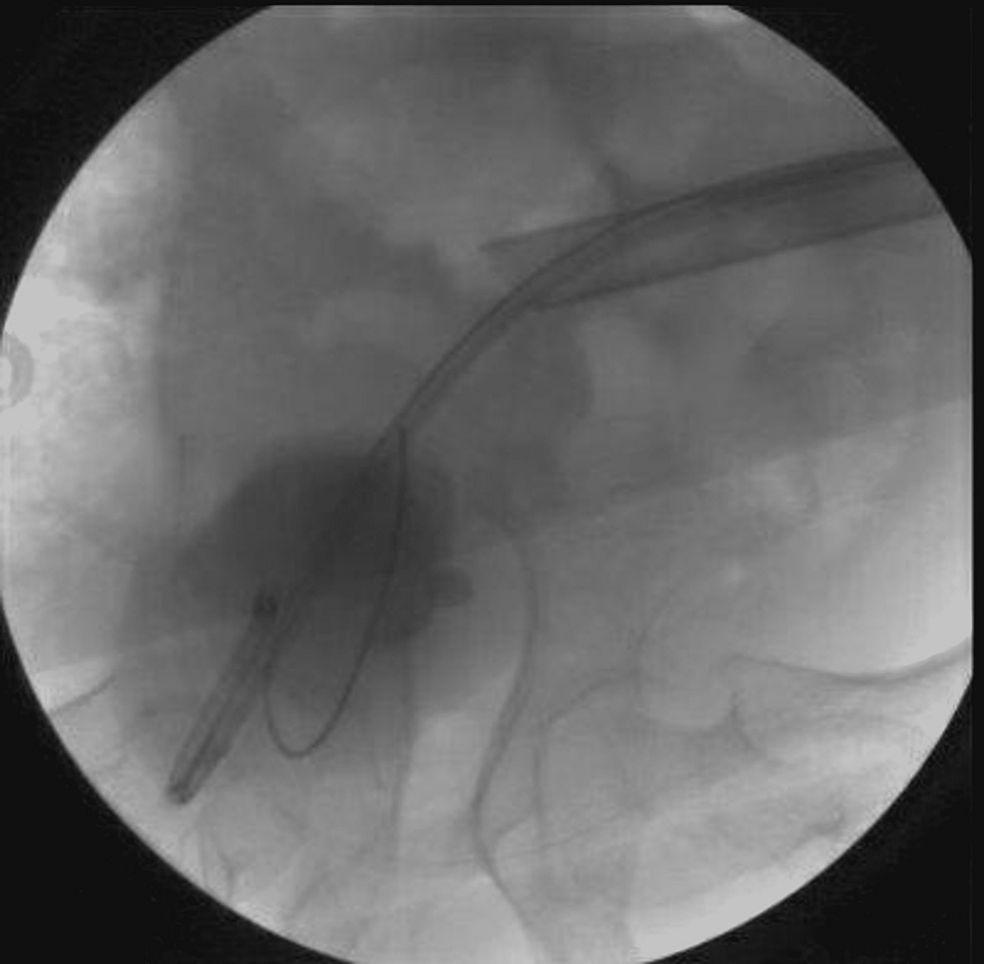
Fluoroscopic image of the ureteroscope advanced into the bladder

Consequently, a nephrostomy balloon dilator was inserted (Figure 4) and a rigid nephroscope was used to inspect the kidney. After that, we switched to a flexible nephoscope. Subsequently, antegrade ureteroscopy was performed, and the stone was seen at the lower ureter. Given the size of the stone, the decision to use a 12/14 access sheath through the nephrostomy sheath was made. Using a 200-um holmium laser fiber, stone fragmentation was done. The ureteroscope was advanced all the way to the bladder to ensure complete stone fragmentation (Figure 5).

**Figure 6:**
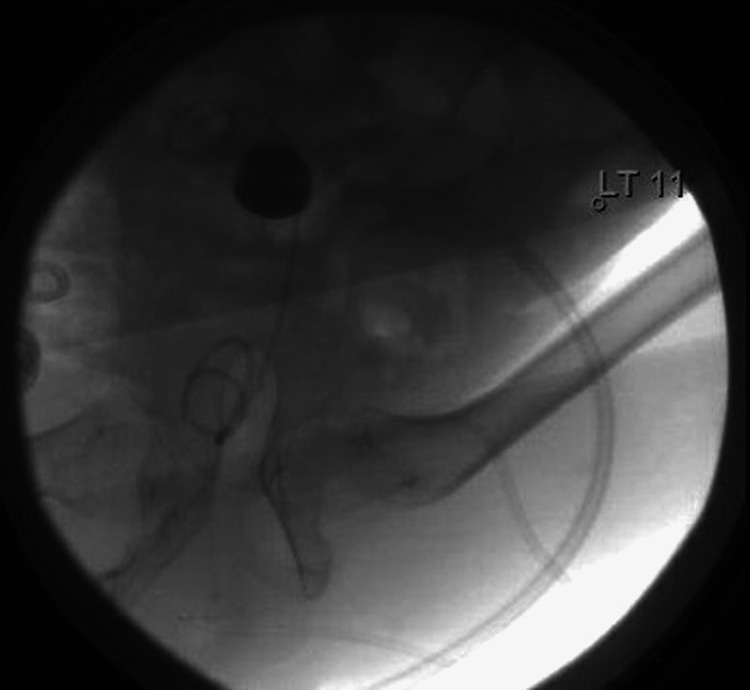
Fluoroscopic image of the inserted 16F nephrostomy tube

In the end, a ureteral stent was placed and a flexible nephroscopy was performed. There were no stones or filling defects. A 16F nephrostomy tube was placed due to the infective nature of the stone and the presence of mild bleeding from the tract (Figure 6).

**Figure 7:**
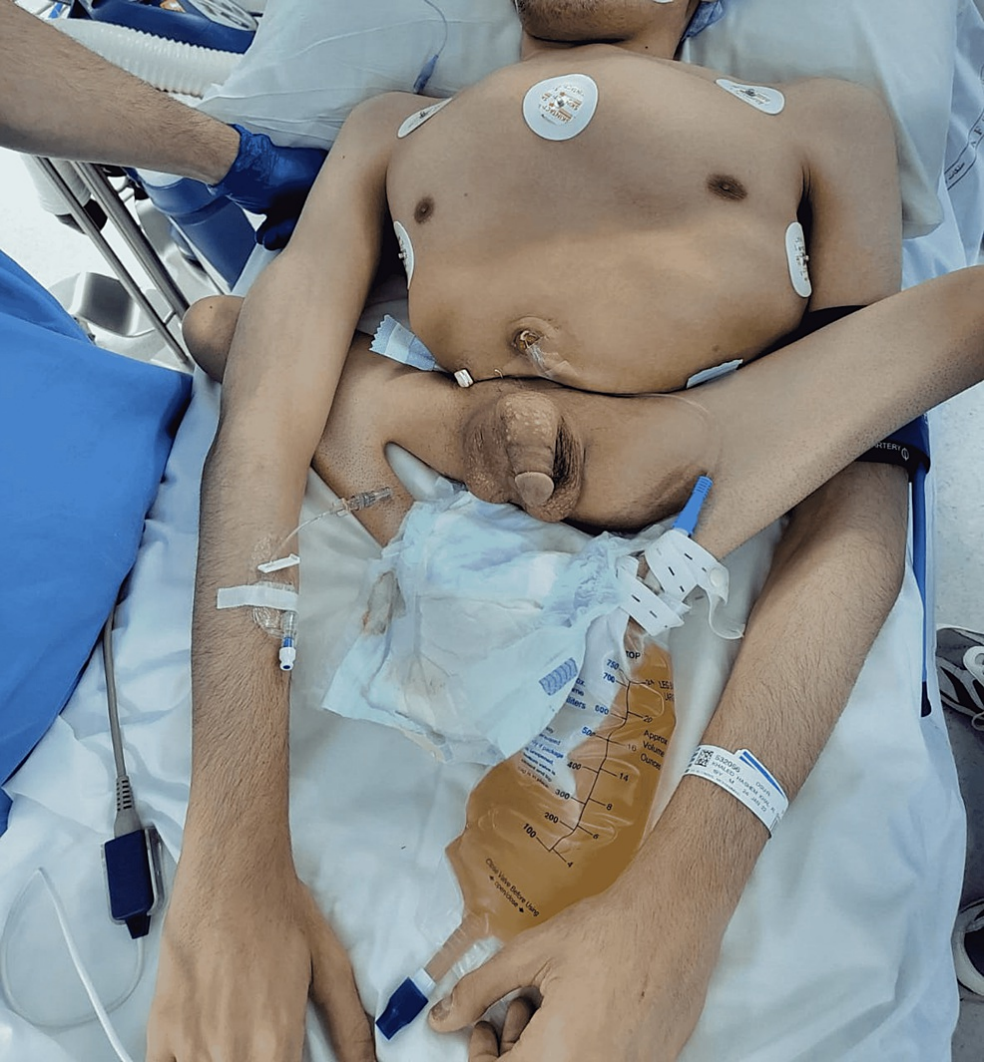
Postoperative image of the patient

We applied pressure dressing at the wound site, and the patient was extubated in a stable condition and was shifted back to a supine position (Figure 7). There were no complications, and the blood loss was only 50 ml. We sent the stone for culture and analysis, which came back positive for Escherichia coli. The patient was admitted for observation and repeated imaging the following day. Our main plan was to control pain and continue antibiotics. The patient was discharged the following day with kidney function improving and flank pain resolving.

## Discussion

CDS or CRS, also known as caudal dysplasia sequence, sacrococcygeal dysgenesis, sacral defect with anterior meningocele, congenital sacral agenesis, sacral regression or dysgenesis, and caudal dysplasia. Two risk factors for this condition include maternal diabetes and family history. Within the first four weeks of embryonic development, abnormal embryologic growth of the caudal mesoderm develops [1]. Many etiologic variables have been proposed, however, the actual pathophysiology of this illness is unknown. In certain situations, maternal alcohol intake or cocaine exposure has been linked. Amino acid imbalances, fetal hypoxemia, and hypo-perfusion or vascular steal hypothesis are some of the other theories explored [1].

The most prevalent abnormalities affecting the CRS group in these studies were reduced renal function (8%-12%), renal agenesis (13%-20%), and neurogenic bladder (60%) [3]. 

Pelvic kidneys are often accidentally discovered since they are usually asymptomatic [4]. One in 2,200-3,000 people is thought to be affected [1]. Despite the fact that they are not normally harmful, they may indicate the development of calices, uteropelvic junction blockage, hydronephrosis, or nephrolithiasis [5]. According to studies, 22% to 37% of individuals with pelvic kidneys also have uteropelvic junction blockage [6]. Uteropelvic junction obstruction is the most frequent prenatally found disease that can lead to hydronephrosis and requires quick diagnosis [7].

As the neurogenic bladder affects 60% of the CRS population [8], all CRS patients should be properly checked for urinary obstruction, especially those who appear with a pelvic kidney, as in this example. Since CRS patients are often asymptomatic until the condition has advanced, utilizing a consistent approach for assessing dysfunctional voiding symptoms is perhaps the most successful method to diagnose them [9].

The PCNL procedure is performed in patients who have kidney stones larger than 2 cm, resistant to other treatments, or in the presence of anatomical abnormalities, which was the indication in our patient [10]. The potential complications of this procedure can be bleeding, infection, and blood loss. PCNL is beneficial in children, with an 83 percent clearance rate and more than 90 percent with dual treatment. When feasible, we recommend keeping tract extension at 24F or less [10]. Percutaneous nephrolithotomy is a safe, effective, and viable therapy option for children with kidney stones that are on average 2 cm in diameter [10].

Although this surgery has shown to be quite safe, there are risks and potential consequences with every surgical operation. When compared to open surgery, the safety and complication rates are comparable. This treatment will result in some blood loss; however, patients will seldom require a blood transfusion. All patients are given broad-spectrum antibiotics to minimize the possibility of infection arising following surgery. Scar tissue might form in the kidney or ureter, necessitating further surgery. Injury to surrounding tissue/organs, such as the bowel, spleen, liver, lung, pancreas, and gallbladder, is a possibility. Kidney failure is uncommon, although it is a possibility. PCNL may be done safely with minimum morbidity in patients with caudal regression syndrome by utilizing adult equipment for significant stone burdens, allowing for rapid and thorough stone removal. Endourologists have distinct hurdles while dealing with stone illness in pelvic ectopic kidneys. In this group of patients, a variety of therapeutic options are available, including laparoscopy, percutaneous methods, ureteroscopy (URS), open surgery, and shockwave lithotripsy (SWL). Although PNL is a widely used treatment option for transplanted and ectopic pelvic kidneys and anatomically normal kidneys, it necessitates a more complex and time-consuming method. The ectopic pelvic kidney, which is positioned anterior to the sacrum and in the retroperitoneum, connects bowel loops between the pelvic kidney and the anterior abdominal wall. A blind percutaneous transperitoneal route to a pelvic kidney should be avoided because of the considerable risk of colon damage [11-14].

## Conclusions

PCNL may be done safely with minimum morbidity in patients with caudal regression syndrome by utilizing adult equipment for significant stone burdens, allowing for rapid and thorough stone removal. The main two obstacles are the complex anatomy and the high intraabdominal pressure, which could be a challenge for the surgeon, interventional radiologist, and anesthesiologist. Moreover, the lack of signs can result in a delay of a neurogenic bladder diagnosis and, eventually, the development of complications such as kidney stones that put a high burden of morbidity on the CRS patient. Thus, urological examinations and careful monitoring are essential in this population.
